# Immunizing with information – Inoculation messages against conversational agents’ response failures

**DOI:** 10.1007/s12525-021-00509-9

**Published:** 2021-12-22

**Authors:** Severin Weiler, Christian Matt, Thomas Hess

**Affiliations:** 1 Institute for Information Systems and New Media, LMU Munich, Ludwigstraße 28, 80539 Munich, Germany; 2 Institute of Information Systems, University of Bern, Engehaldenstr. 8, 3012 Bern, Switzerland

**Keywords:** Conversational agent, Chatbot, Inoculation messages, Elaboration likelihood model, Customer service, C91, D91, L86, M31

## Abstract

Conversational agents (CAs) are often unable to provide meaningful responses to user requests, thereby triggering user resistance and impairing the successful diffusion of CAs. Literature mostly focuses on improving CA responses but fails to address user resistance in the event of further response failures. Drawing on inoculation theory and the elaboration likelihood model, we examine how inoculation messages, as communication that seeks to prepare users for a possible response failure, can be used as an alleviation mechanism. We conducted a randomized experiment with 558 users, investigating how the performance level (high or low) and the linguistic form of the performance information (qualitative or quantitative) affected users’ decision to discontinue CA usage after a response failure. We found that inoculation messages indicating a low performance level alleviate the negative effects of CA response failures on discontinuance. However, quantitative performance level information exhibits this moderating effect on users’ central processing, while qualitative performance level information affected users’ peripheral processing. Extending studies that primarily discuss ex-post strategies, our results provide meaningful insights for practitioners.

## Introduction

Conversational agents (CAs) are dialogue systems that interact with users through natural language. They have been described as having “humanlike behavior” (Vassallo et al., 2010), transforming information systems (IS) usage interactions to resemble human-to-human encounters (Maedche et al., 2019). CA technology has seen much improvements over the past decade (Keyser et al., 2019), with positive results, such as service satisfaction (Diederich et al., 2019d) and enjoyment (Lee & Choi, 2017), but response failures remain an ongoing issue for CA acceptance (Følstad & Brandtzæg, 2017).

CA response failures are responses to user requests that are not meaningful, stemming from inadequate knowledge bases or a failed natural language understanding and/or processing (Adam et al., 2021; Diederich & Brendel, 2020). Response failures affect many CAs with varying levels of maturity and are not limited to poor- quality CAs. Among other things, response failures can be an inherent consequence of the underlying, self - learning system components, which first require certain amounts of data from a user to be able to provide adequate personalized support to that user (Diederich & Brendel, 2020). In the event of a CA response failure, users often question its capabilities and loose trust in the system. Often CA response failures lead to an immediate discontinuance of CA usage, giving the system no chance “to make it up” to the user (Ben Mimoun et al., 2012; Luger & Sellen, 2016). The risk of response failures impacting the success of CAs is underlined by the fact that numerous corporations, IKEA (Ben Mimoun et al., 2012) and Facebook (Orlowski, 2017) among them, shut down their CAs following high response failure rates. In the case of Facebook, a response failure rate of about 70% was reported for its CA “M” (Weinberg, 2017).

Despite the noted performance deficiency of CAs, little research has been done on how to overcome users’ discontinuance following a CA response failure. CA research focuses on the role of social presence (Diederich et al., 2019a; Zhang et al., 2012) and anthropomorphic CA features (Derrick et al., 2011; Elkins et al., 2012) or other design cues and their impact on human behavior with regard to perceptions and adoption (Diederich et al., 2020; Qiu and Benbasat 2009). With only five notable exceptions (Ben Mimoun et al., 2012; Diederich & Brendel, 2020; Diederich et al., 2020, 2021; Luger & Sellen, 2016), IS research has investigated CAs in mostly failure-free scenarios. In real life, however, situations often arise where the limited performance of a CA results in an abrupt response failure (Følstad & Brandtzæg, 2017).

While enhancing CA performance seems an obvious condition for increasing user acceptance, it is still necessary to take measures to prevent users’ immediate discontinuance of the CA (Adam et al., 2021; Diederich et al., 2020). Since the presentation of IS has significant effects on subsequent user behavior, an IS’ presentation can be used to counteract usage discontinuance (Becker et al., 2020). A promising approach in this context is inoculation theory, which postulates that individuals can be made to resist attitude-changing messages or events if they have previously been prepared for their occurrence. This immunization is generally conveyed via inoculation messages that contain a forewarning to the individual, and a refutational argument followed by a supportive pretreatment. While being largely neglected in IS research, inoculation messages have been successfully applied to areas such as health, politics and marketing, spanning decades of theoretical and empirical (laboratory and field) research (Banas & Rains, 2010; Compton, 2012).

Inoculation messages affect individuals’ expectations by alerting them explicitly to a threat while at the same time refuting the negative impact on an outcome. Just as a flu shot preemptively protects against the pathogen by exposing a person to a weakened flu virus, an inoculation message preemptively protects against stronger stimuli by forewarning and offering a refutational pretreatment. This approach is particularly suitable for CA response failures, as CAs are regularly expected to perform better than they actually do (Diederich et al., 2019c; Følstad & Brandtzæg, 2017; Luger & Sellen, 2016). We investigate how the presentation of an inoculation message prior to a CA response failure affects users’ actual discontinuance behavior, making them immune to the CAs’ intermittent underperformance. Hence, our research is guided by the following research question **(**RQ**)**: *Can inoculation messages alleviate users’ actual discontinuance in the event of a CA response failure?*

Inoculation messages as an immunization strategy are most effective if they trigger users’ internal counterarguing, which is the raising and refuting of arguments about the issue at hand, in addition to the stated arguments (Compton & Pfau, 2005). In the context of a CA response failure, an event of underperformance, inoculation messages need to address the CA’s performance in advance to leverage an immunizing effect. The performance information needs to be designed carefully to preclude any unintended effect on users’ processing of the response failure. According to the elaboration likelihood model (ELM), users can change attitudes via a central processing or peripheral processing of information given. In light of this, our study examines the effect of the CA’s *performance* and the *linguistic form* of the performance information on users’ actual discontinuance. We conducted a randomized online experiment based on an artificial intelligence (AI) and text-based CA (i.e., a chatbot), which is a particular type of CA designed for turn-by-turn text-based conversations with human users (Adam et al., 2021). More specifically, we varied the content of a two-sided inoculation message, a special type of inoculation message (see section *Inoculation Theory)*, and determined the subsequent effects on our dependent variable, actual discontinuance.

To our knowledge, this is the first empirical study that examines and explicitly compares the effectiveness of different inoculation messages in alleviating detrimental effects of CA response failures. This study contributes to the literature on the ELM by examining how inoculation messages are processed and how they influence usage behavior. It also fills a void in existing research on CAs by shifting the focus from improving the technical capabilities of CAs, for which a level of perfection might not be reached any time soon, to the investigation of CA response failures and users’ reactions to them. Lastly, by offering an evaluation of a cost-effective mechanism that fosters continuous usage of imperfect CAs, this study contributes to the successful diffusion of CAs at multiple interfaces, to automate processes and make information more accessible.

## Theoretical background

### The effects of CA response failure and their alleviation

A majority of CAs are implemented as part of customer service and after-sales because CAs promise to address a key challenge, the balancing of service efficiency and service quality. CAs are intended to save costs for providers (Gnewuch et al., 2017), while enhancing customer experience by offering fast and convenient 24/7 support (Scherer et al. 2015). CAs are a reality in electronic markets and customer services on many websites, social media platforms and in messengers (Nguyen et al., 2018). This is underscored by the increase of active Facebook chatbots from 11,000 in 2016 to 300,000 in 2019 (Facebook, 2019). Despite this promising outlook, current CAs are mostly limited in terms of their usage of machine intelligence and overall system capabilities (Diederich et al., 2020), resulting in a lack of sufficient conversational capabilities (Følstad & Brandtzæg, 2017). As a consequence, user interactions with CAs have been described as unconvincing or frustrating and marked by response failures (Diederich et al., 2020; Knijnenburg & Willemsen, 2016). CA response failures are the inability to provide a meaningful reaction to user requests (Diederich & Brendel, 2020) and technically stem from a constrained knowledge base, failure in natural language processing or other technical glitches (such as misaligned interfaces). CA response failures become apparent to the user when responses are misrouted or through fallback messages (e.g., “Sorry, I didn’t get that.”).

Response failures are particularly problematic because they often result in users discontinuing their usage. The risk of discontinuance following a CA response failure is substantially increased by unrealistic user expectations of what the CA is objectively capable of (Ben Mimoun et al., 2012; Diederich & Brendel, 2020). Knijnenburg and Willemsen (2016) showed that users of CAs rich in anthropomorphic features overestimated the system, tried to exploit capabilities that were not signaled and subsequently discontinued their usage. Additionally, unrealistic user expectations are amplified by the fact that contemporary CAs are very good at creating a high level of social presence through anthropomorphism (Ben Mimoun et al., 2012)^1^, defined as “the attribution of human-like (physical or non-physical) features, behaviors, emotions, characteristics, and attributes to a non- human agent or an inanimate object” (Maedche et al., 2019, p. 538).

Studies on the alleviation of negative effects of CA response failures, have examined how politeness and apologetic behavior on the part of the CA alleviates users’ emotional reactions after a response failure (Lee et al., 2010; Medhi Thies et al., 2017). Other scholars have explored repair strategies to facilitate the completion of tasks left unfinished because of response failures, such as offering alternative answer options or further explanations (Ashktorab et al., 2019; Følstad & Taylor, 2020). However, this literature focuses on ex-post alleviation measures of the negative consequences of CA responses, ignoring the risk of users’ immediate discontinuance after a CA response failure. Hence, to the best of our knowledge, the problem of preventively, or preemptively, addressing CA response failures and thus ensuring that users do not discontinue their usage has not been addressed. A promising and, to date, unrecognized approach in IS research to solve this problem is the use of inoculation messages.

### Inoculation theory

The design of inoculation messages is based on the principles of inoculation theory, in which individuals are preemptively influenced not to change their mind after receiving a stimulus that challenges their status quo attitude. According to inoculation theory, individuals can be protected against persuasion or influence (i.e., attempted attitude change) through messages, just as they can be immunized for viruses through a medical inoculation (McGuire, 1964). Consider the example of a flu shot, which consists of a flu antigen weak enough not to make the individual sick, but strong enough to trigger the production of antibodies that create immunity. In the context of resistance to attitude change, inoculation messages that are weakened versions of arguments or events – again, weakened to prevent attitude change, but strong enough to enable protective responses – are presented to the individual. Inoculation messages are intended to trigger a cognitive process of *counterarguing* (Banas and Miller 2013), which involves the raising and refuting of arguments about the issue in addition to the already stated refutational component, by the individuals themselves (Compton, 2012).

Inoculation messages come in different forms. However, the most established two forms are one- and two-sided inoculation messages. One-sided messages expose the receiver to a potential threat, thus serving as a *forewarning* (Pfau, 1995), and are a necessary component of any inoculation message (Ivanov et al. 2013; McGuire, 1964). To stick with a medical analogy, an example of a one-sided message is a message that claims the spread of a potentially harmful virus. Two-sided inoculation messages adopt the threat component from one- sided messages, adding a contrasting, or competitive, element (O’Keefe, 1999). This contrasting element can be formulated as a *supportive* and/or a *refutational pretreatment* (Compton, 2012). A supportive pretreatment against the threat would be, in our example, promoting a specific vitamin regimen as a way to boost health before encountering the virus. A refutational pretreatment would, on the other hand, put forward persuasive challenges to the threat, such as contesting the potential harm of the novel virus. Generally, stand-alone one- sided messages have less credibility than two-sided messages and are quickly discounted by viewers (Allen, 1991). Two-sided messages are rhetorically superior because they encourage greater scrutiny from receivers (O’Keefe, 1999).

### Inoculation messages to reduce users’ discontinuance

Inoculation messages generally convey resistance to influence and persuasion (Compton & Ivanov, 2012), and are not limited to persuasion attempts in written form, as they are effective against non-written attacks, such as photographs (Pfau et al., 2008) or videos (Dillingham & Ivanov, 2017). Inoculation messages first gained general interest when they proved to be effective for an antismoking campaign: Inoculation messages helped children to resist the pressure to smoke (Pfau et al., 1992), and proved to induce attitude changes which lasted up to 20 months after the inoculation (Pfau & van Bockern, 1994). Another classical field of application for inoculation theory is political communication. While preempting attacks on candidates’ images and their positions, inoculation messaging offers an approach to protecting and thus securing votes (Compton & Ivanov, 2013). Beyond health and politics, inoculation messages were applied in the fields of corporate communication, public relations and marketing (Compton, 2012). Inoculation messages contribute to corporate identity formation, commitment, consciousness, and sportsmanship within organizations (Haigh & Pfau, 2006). Pre-crisis communications based on inoculation messages also protect an organizations’ reputation following a crisis (Wan & Pfau, 2004). In marketing, inoculation messages mitigate the influence of competitors’ comparison ads (Ivanov et al., 2009) or increased consumers ’ resistance to marketing (Compton & Pfau, 2004).

In the IS literature, inoculation messages were sparsely used, improving the retention of information technology (IT) professionals (Fagnot & Stanton, 2015), or of consumers’ IT buying behavior (Goethals et al., 2012). Recently IS research has acknowledged the importance of inoculation messages by integrating them in their research approaches. Their interim findings show that inoculation messages could be an effective remedy of fake news (Patel & Constantiou, 2020) and point out that they could be used to prevent phishing (Wu et al., 2020).

**Fig. 1:**
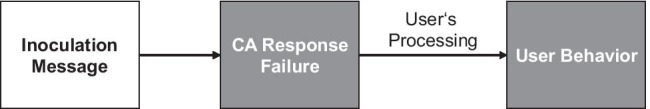
User’s processing of CA response failures after inoculation

To ensure that users do not change their opinion about the CA (i.e., discontinuance) after a response failure, a two-sided inoculation message can be applied (see Fig. 1). In the case of a CA response failure, the *forewarning* of the inoculation message should make users aware of the CAs’ performance deficit. The *supportive pretreatment* should offer a cue to bypass the threat, while the *refutational pretreatment* should refer to the CAs’ performance and downplay the severity of the CA response failure. In this way, the refutational pretreatment relativizes the failure and reassures the user. As inoculation messages are most effective when they trigger users’ internal counterarguing, all message components need to be evaluated against the intended effect, which, in the case of a CA response failure, is to make users immune to the CA’s underperformance. While the design of the forewarning as well as the supportive pretreatment does not leave much margin, the CA performance information itself remains most important for assessing the effectiveness of the inoculation message. The performance of a CA is typically evaluated based on the relative proportion of correctly handled user requests. A success rate can assume a quantitative (e.g., 98%) or a qualitative form (e.g., almost all).

Therefore, the two content design characteristics are the content of the information (i.e., the *level of performance*) and the presentation of the performance information (i.e., the *linguistic form* of performance information), which need to be configured for optimal inoculation.

### Processing of CA response failures

To understand how users process technology-mediated information IS researchers have frequently applied the elaboration likelihood model (ELM), making it a well-established model of informational influence (Sussman & Siegal, 2003; Tam and Ho 2006). The ELM postulates that the formation of an individual’s attitudes following a persuasion attempt takes place through two distinct cognitive processes: central processing (also called central route) and peripheral processing (peripheral route) (Petty & Cacioppo, 1981, 1986).

Central processing demands a high level of elaboration, while peripheral processing demands a low level of elaboration. When a message is processed centrally, the aspects presented in the message are carefully examined and the arguments are evaluated. Processing peripherally implies the usage of simple heuristic cues or informational indicators. According to the ELM, the route of information processing is determined by the elaboration likelihood, which is a temporal state that is influenced by an individual’s cognitive ability and motivation to evaluate the key merits of a focal object (Bhattacherjee & Sanford, 2006). To activate central processing, the individual's motivation and cognitive abilities must be strong enough to ensure that a high level of elaboration is achieved. The cognitive abilities necessary to achieve high levels of elaboration are supportive working memory and effective retrieval of relevant information from long-term memory. In contrast, peripheral processing depends on the availability of cues and heuristics related to emotions and intuition. Further, it is independent of the working memory and cognitive ability. Furthermore, peripheral processes are fast, automatic and unconscious, whereas central processing is slow, controlled and conscious (Evans, 2011). Both types of processing are independently accessed, with central processing being influenced by peripheral processing only rarely (Evans, 2011).

IS researchers have used the ELM to describe how attitudes about information generated by an IS are processed (e.g., Aghakhani et al., 2020; Cheung et al., 2012), paralleled with a second stream in which the theory was applied to understand the emergence of attitudes toward a system itself,, such as download intention or trust in mobile banking (e.g., Gu et al., 2017; Zhou, 2012). Building on the latter and ELM’s suitability to distinguish different forms of cognitive processes, we apply the ELM to understand whether and how the presentation of inoculation messages influences the formation of attitudes and discontinuance behavior about a CA after response failures. Therefore, we identify the most important determinants of user interaction depicted in previous CA literature (Zierau et al., 2020). Especially important for individual-level adoption of CAs are productivity-related variables, representing the utility of CAs for users, which can help reduce their workload. Since a cost-benefit analysis requires large cognitive capacities, this evaluation takes place on users' central processing route (Kroenung & Eckhardt, 2017). CAs are designed to efficiently perform specific tasks for users, thus their productive benefit is expressed by the user’s perceived performance (Eren, 2021; Pizzi et al., 2020). In addition, user trust was identified as being crucial for the adoption of CAs (Araujo, 2018; Laumer et al., 2019; Prakash and Das 2020; Zierau et al., 2020) stemming from the fact that the usage of a CA entails the delegation of parts of a task from the user to the CA (Jung et al., 2018a, 2018b). Delegation manifests an agency relationship, as the CA takes responsibility from the users (Bergen et al., 1992). This agency relationship is characterized by information asymmetry, as the CA has access to more information than the user regarding the target behavior (e.g., how the CA is handling tasks). Without such information, the user cannot fully verify the abilities and skills of the CA and thus needs to rely on heuristics, implying a peripheral processing (Wang & Benbasat, 2007). All in all, CA response failures cause users to reassess the performance of a CA (central processing) and to adjust their trust in the system (peripheral processing) providing a lever for the alleviation of the negative effects of CA response failures.

## Hypotheses development

### Main effects

**Fig. 2:**
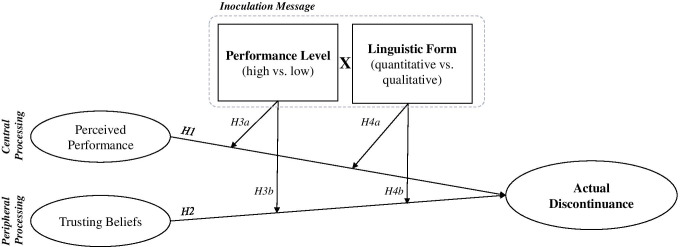
Conceptual model

We apply the ELM to understand how inoculation messages, communicated through a CA, are processed by users and what effects they have on users’ discontinuance after a CA response failure (see Fig. 2).

The instrumentality of system use is an important reason why individuals continue their usage of a system (Islam et al., 2017). A system is instrumental to users if they perceive its performance as good and if they believe that continued use will lead to future gains in utility (Kim et al., 2009). As demonstrated in previous research, users’ intention to continue to use an IS will be determined by their beliefs about performance based on previous system use (Bhattacherjee, 2001; Venkatesh et al., 2011). This is because as attitudes trigger independent and correlated behavioral responses, the central variable of perceived performance determines users’ usage behavior (Venkatesh et al., 2003). Hence, in the case of a response failure, we predict that the more a user believes that a system has performed well, the less likely the user’s actual discontinuance of CA usage:

H1: Perceived performance will reduce users’ actual discontinuance after a CA response failure.

Like central processing, peripheral processing also takes place in the case of a CA response failure. Especially in the early adoption phase, trust is a major determinant of usage behavior, as users tend to have limited usage knowledge on which to base their behavior (Qiu and Benbasat 2009). This is in line with prior research that has found that positive experiences with trustworthiness increases the intention to trust (Benbasat & Wang, 2005; Venkatesh et al., 2016). Hence, we predict that the more trust users have in a CA, the less likely they will be to discontinue usage after experiencing a response failure. Thus, we hypothesize:

H2: Trusting beliefs will reduce users’ actual discontinuance after a CA response failure.

### Moderating effects

Generally, CA response failures provoke negative reactions from users (Xu et al., 2014). According to inoculation theory, individuals can be inoculated against threats, or made resistant to an attitude change, through inoculation messages (Compton, 2012). As described above, to promote resistance to attitude change after a CA response failure, inoculation messages need to include (a) information about the CA’s performance because the refutational pretreatment section of the inoculation message should refer to the potential threat to the user. In addition, the performance information needs to be (b) presented in the optimal linguistic form.﻿ 

(a) In the case of a CA response failure, the potential threat to the user is the CA’s underperformance. Based on this and given the basic functionalities of most contemporary platforms for CA design^2^, this performance information needs to be presented to the user in written form. CAs vary in terms of their declared performance level, depending on the communication mode (e.g., text-based) or the context (general purpose vs. domain specific) (Diederich et al., 2019a). A low performing CA is likely to result in a greater number of CA response failures. However, response failures in both high performing and low performing CAs can’t be avoided completely since they are sometimes caused by factors beyond the provider’s control (e.g., typos). In the case of a response failure for high performing CAs, the use of inoculation messages carries the risk of a user distrusting the CA’s credibility (Hwang & Jeong, 2016). According to the discounting hypothesis, the credibility of the sender of a message is discounted if this information later proves to be unreliable (Allen, 1991; Smith, 1982; Allen and Stiff, 1989). Therefore, if the performance level promised in the inoculation message does not match the actual performance, users may react negatively to the message, which increases the likelihood that they will discontinue using the CA after a response failure. Hence, we hypothesize that an inoculation message for a CA with a low declared performance level alleviates the effect of perceived performance/trust on actual user discontinuation to a greater extent than would be the case for a CA with a high declared performance level:

H3a: Compared to an inoculation message declaring a high performance level, a message for a CA with a low declared performance level alleviates the effect of perceived performance on actual user discontinuation to a greater extent.

H3b: Compared to an inoculation message declaring a high performance level, a message for a CA with a low declared performance level alleviates the effect of trusting beliefs on actual user discontinuation to a greater extent.﻿

(b) A CA performance level is a ratio indicating the relative proportion of correctly handled user requests, and, in the case of text-based CAs, the performance level needs to be presented in written form. To express a CA’s performance level, either a quantitative (e.g., 98%) or qualitative (e.g., almost all) linguistic form can be used. A large amount of research has been conducted to observe individuals’ cognitive evaluations and behaviors after exposure to information with varying narrative structures and pictorial designs, as well as varying quantitative vs. qualitative cues (Childers & Viswanathan, 2000; Evans & Stanovich, 2013; Jiménez-Barreto et al., 2021). Despite this research and the recognition that a quantitative linguistic form promotes more accurate decisions than a qualitative one, they are often used interchangeably (Liu et al., 2020, 2021). The differences between the two stem from different mental processes. A quantitative linguistic form is processed more intentionally and analytically than a qualitative one, taking up more mental resources and thus corresponding to the central route of information processing of the ELM (Ayal et al., 2015; Liu et al., 2020; Tzelgov et al., 1992). The use of quantitative performance information within an inoculation message will activate elaborate mental processing, leading to a thoughtful consideration of the message and the subsequent decision (Childers and Viswanathan 2000; Liu et al., 2021). Therefore, users will be convinced by the line of reasoning behind the inoculation message to continue using the CA. This impact on their discontinuance propensity is expressed in an alleviating effect of the inoculation message on the central route of information processing. Hence, we hypothesize that a quantitative linguistic form of the performance information will moderate the effect of the perceived CA performance on users’ actual discontinuance:

H4a: A quantitative linguistic form will moderate the effect of perceived performance on user’s actual discontinuance.

In contrast to a quantitative linguistic form, a qualitative linguistic form is processed more intuitively, activates fewer mental resources, and thus corresponds to ELM’s peripheral route of information processing (Ayal et al., 2015; Liu et al., 2020; Tzelgov et al., 1992). A qualitative linguistic form causes a lower level of analytical engagement with the information provided, resulting in users not carefully reflecting on the argumentation within the inoculation message (Petty et al., 1981). However, users can still be influenced by a qualitative linguistic form, as the mere presence of meaningful information or explanations about the causes of a certain event can signal the provider’s competence to users (Komiak et al., 2004; Wang & Benbasat, 2008). Therefore, the qualitative form within an inoculation message can mitigate the negative effect of CA response failures by having an alleviating effect on the peripheral route of information processing. Hence, we hypothesize that aqualitative linguistic form of performance information will moderate the effect of trusting beliefs on users’ actual discontinuance:

H4b: A qualitative linguistic form will moderate the effect of trusting beliefs on user’s actual discontinuance

## Methodology

### Experimental design

We used a 2x2 (plus control group) between-subjects experimental design that isolated individual and interaction effects on users’ actual discontinuance (Adam et al., 2021; Schneider et al., 2020) . The experiment was a randomized online experiment in the context of a customer-service chatbot for online banking that handled customers’ banking requests (e.g., transfers, balances and credit card payment due dates). We chose digital banking because it has been used as a generalizable case in previous IS research on, for example, automation and recommendation systems (Kim et al., 2018) and compliance with CAs (Adam et al., 2021). Moreover, it is a context that is seen as a fruitful application area for CAs because they have the potential to cost-effectively automate a lot of common user requests (e.g., checking account balances, blocking credit cards, etc.) as they are based on routine tasks (Jung et al., 2018a, 2018b; Jung et al., 2018a, 2018b).

In our experiment, we employed a self-developed text-based CA that replicated the design of many contemporary chat interfaces (see Appendix for a screenshot of the chatbot interaction interface). The users could interact with the CA by typing in their message in natural language and pressing enter or by clicking the Send button. We developed our CA on the basis of the prebuilt banking agent from Google’s Dialogflow CA design platform (Google, 2020). The Dialogflow platform provided us with the necessary AI-driven capabilities for understanding natural language processing and dialogue management. The AI in the Dialogflow cloud processed, understood, and answered the input that was freely entered into the chat interface by the participants, enabling them to use their own words. Therefore, our CA worked in the same natural manner and with similar capabilities as other contemporary AI applications, like IBM Watson Assistant, Amazon’s Alexa and Apple’s Siri, albeit in written form and narrowed down to the specific tasks of the experiment. Further, we limited the anthropomorphic features of the chatbot (e.g., an average response time of two seconds indicated by a “typing” icon) to a minimum, mimicking a situation where the mismatch between the anthropomorphic capabilities and the conversational capabilities was marginal. We assumed that in situations where this mismatch was greater than in our study, discontinuance rates would be even higher because of a “surprise effect” that takes place when users find out that they are interacting with a machine, despite having had a human-like experience prior to the CA response failure (Diederich et al., 2020).

### Manipulations

All participants were presented with the same dialogues and were asked to solve the same tasks. The treatment groups were presented with two-sided inoculation messages, with which we manipulated two performance information variables: (a) the *level of the performance* information and (b) the *linguistic form* of the CA performance information (see Table 1).

**Table 1:** Treatment groups

	**Two-sided Inoculation Messages**				**No inoculation**
**Performance Level**	Low		High		Control group
**Linguistic Form**	Quantitative	Qualitative	Quantitative	Qualitative	-
Treatment Group	a	b	c	d	e
Expression	“60%”	“a lot”	“98%”	“almost all”	-

**Fig. 3:**
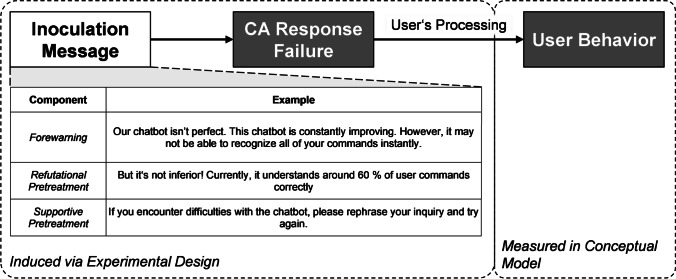
Measurement scheme and types of inoculation messages

The two-sided inoculation messages consist of two subsequent messages (see Figure 3). First, message receivers are given a forewarning, providing them with a pre-taste of an actual threat that they might experience later (Compton & Pfau, 2004; McGuire, 1964). In our study, we told participants that the CA might be imperfect and that response failures could occur: “Our chatbot isn’t perfect. This chatbot is constantly improving. However, it may not be able to recognize all of your commands instantly.” Second, the potential threat needs to be downplayed with a refutational pretreatment. This can be achieved by offering the user a carefully constructed counterargument, or competitive frame (O’Keefe, 1999). We constructed a refutational paragraph, building on the CA’s performance information: “But it’s not inferior! Currently, it understands around [*treatment*] % of user commands correctly.” We opted to use performance information in the refutational paragraph because the potential threat (i.e., CA response failure) would be the result of a lack of performance, hence, refuting the threat entailed providing counterarguments such as an objectification of the CA’s performance. The second message was rounded off with a supportive pretreatment, aimed at convincing the participants to put more effort into formulating their requests: “If you encounter difficulties with the chatbot, please rephrase your inquiry and try again.”

The refutational pretreatment entailed our treatment variables (linguistic form of performance information and level of performance information). We varied the linguistic form of the performance information by giving a quantitative quantifier (a percentage of the CA’s interaction success rate) or a qualitative quantifier (a verbal equivalent of the quantitative quantifier). For the low performance level, we chose quantitative and qualitative quantifiers: (a) 60% and (b) “a lot.” For the high performance level, we chose (c) “98%” as a quantitative quantifier and the words (d) “almost all.” In the (e) fifth treatment group, we didn’t show the participants any message (i.e., *no inoculation*).

### Procedure

**Fig. 4:**
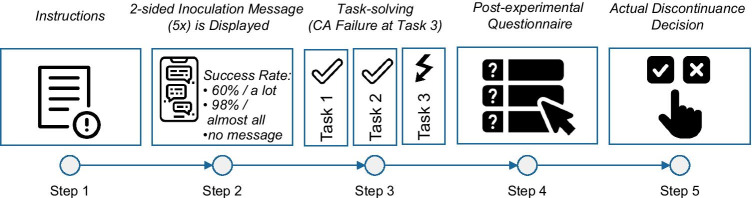
Experimental procedure

We set up the participants in a customer service scenario in which they were supposed to solve three different tasks in collaboration with the banking CA (see Appendix for detailed dialogue flows and instant messenger interface). The experimental procedure consisted of five steps (see Figure 4).

- (1) We presented a short introduction to the participants that included information about the experimental procedure and instructions for the task-solving. We told participants that the entire study consisted of six banking tasks and that after half of the tasks had been completed, they would be asked to complete a questionnaire. After the questionnaire, they would have to decide whether to stay with the CA for the second half of the experiment or switch to traditional online banking (navigating through the bank’s online banking web interface). In actuality, the experiment ended after the questionnaire, but we used this dissimulative design because we wanted to capture participants’ actual discontinuance decisions rather than only statements of intent. The incentive structure was entirely dependent on the participants’ performance during the task-solving (26 pence/task, 1.56 pounds maximum in total), without the guarantee of a basic payoff﻿.
- (2) Participants were randomly allocated to one of five inoculation message treatments. All messages were two-sided inoculation messages that consisted of two paragraphs, one forewarning and one pretreatment paragraph, with the exception of the control group, which received no inoculation message. The content of the message was displayed in a single page before the task-solving began and was repeated as part of the CA’s welcome message during the task-solving to ensure that participants noticed it.
- (3) Next, participants were asked to solve three different banking tasks. The tasks were timed with 150 seconds for task 1 and 90 seconds for tasks 2 and 3 (see Appendix for a detailed description of exemplary dialogues for the different tasks). The chatbot worked properly for the first two tasks (balance inquiry, money transfer). However, the CA’s knowledge base was modified to be unable to give the participants the information necessary to complete the third task (inquiry of credit card billing due date). Any effort to retrieve the required information resulted in a fallback message (e.g., “Sorry, I didn’t get that”).
- (4) After the completion of the third task (either by running out of time or by clicking “next”) participants answered the post-experimental questionnaire about their CA experience and other questions, capturing our independent and control variables.
- (5) Finally, participants could decide whether to continue the second half of the task-solving with the CA or switch to traditional online banking with a web interface (this was the basis for the dependent variable).

After capturing their decisions, we debriefed the participants, thanked them for their participation and guaranteed them the maximum payoff, regardless of their performance during the task-solving.

### Operationalization

We measured our dependent variable, actual discontinuance, as a binary coded variable, asking our participants to choose between the two options: “I will continue with the chatbot.”, “I will continue in the traditional way (Bank's website)”. Our independent variables, perceived performance and trusting beliefs, were adapted from Kim et al. (2009) and Benlian et al. (2012) respectively, and measured on a 7-point-Likert-type scale ranging from strongly disagree to strongly agree (see Table 2).

**Table 2:** Measurement scales

Variables	Wording	Reference
*Perceived* *Performance*	1. Using the Chatbot improved my performance in the tasks 2. Using the Chatbot increased my productivity in the tasks 3. Using the Chatbot enhanced my effectiveness in the tasks 4. Overall, using the Chatbot is useful for the tasks	Kim et al. (2009)
*Trusting Beliefs*	1. The Chatbot was competent in supporting the completion of the tasks 2. The Chatbot performed its role of supporting the task completion very effectively 3. Overall, the Chatbot supported me to solve my tasks 4. I believe that the Chatbot’s dealings with me were in my best interest 5. The Chatbot’s dealings with me felt like it would do its best to help me 6. I believe that the Chatbot’s was truthful to me 7. I would characterize this type of Chatbot’s dealings with me as honest 8. The Chatbot appeared to be unbiased 9. The Chatbot is sincere and genuine	Benlian et al. (2012)
*User’s Expertise*	1. I felt very competent in the previously assigned tasks 2. I felt able to meet the challenge of performing well in the previously assigned tasks 3. I was able to master the previously assigned tasks 4 .I was good at doing the previously assigned tasks	Tauchert and Mesbah (2019)
*Degree of* *Realism*	The use of the chatbot was realistic	Adam et al. (2021)
*Personal* *Innovativeness*	1. If I heard about a new information technology, I would look for ways to experiment with it 2. Among my peers, I am usually the first to try out new information technologies 3. In general, I am hesitant to try out new information technologies 4. I like to experiment with new information technologies	Agarwal and Prasad (1998)
*CA Usage*	How often do you use chatbots?	Adam et al. (2021)
*Propensity to* *Trust*	1. It is easy for me to trust a person or an object 2 .My tendency to trust a person or an object is high	Cheung and Lee (2002)
	3. I tend to trust a person or an object, even though I have little knowledge of it	Hampton- Sosa and Koufaris (2005)
*Need for* *Cognition*	1. I would prefer complex to simple problems 2. I like to have the responsibility of handling a situation that requires a lot of thinking 3. Thinking is not my idea of fun 4. I would rather do something that requires little thought than something that is sure to challenge my thinking abilities 5. I really enjoy a task that involves coming up with new solutions to problems 6. I would prefer a task that is intellectual, difficult, and important to one that is somewhat important but does not require much thought	Coelho et al. (2018)

All variables, except for *CA Usage*, were measured using an ordinal seven-point Likert scale (from 1 = strongly disagree, 4 = neutral, to 7 = strongly agree). All points of the scale were labeled

In addition to our dependent and independent variables, we also tested demographic factors (age and sex) and the control variables that have been found in the literature to be relevant (see Table 2). We measured propensity to trust (Cheung & Lee, 2002; Hampton-Sosa & Koufaris, 2005), personal innovativeness (Agarwal and Prasad 1998), need for cognition (Coelho et al., 2018), user’s expertise (Tauchert & Mesbah, 2019), and participants’ perceived degree of realism of the CA usage (Adam et al., 2021) on a 7-point Likert-type scale with anchors ranging from strongly disagree to strongly agree. We also determined how regularly participants used CAs, on a 7-point scale with anchors ranging from daily to never (Adam et al., 2021). We included control variables in the model if they showed a significant Pearson correlation coefficient with the dependent or independent variables (Frost, 2019).

All scales exhibited satisfying levels of reliability (α > 0.7) (Han & Yang, 2018; Ko et al., 2005). A confirmatory factor analysis also showed that all the scales we analyzed exhibited satisfactory convergent validity. Furthermore, the results revealed that all discriminant validity requirements were met, since each scale’s average variance extracted exceeded multiple squared correlations (Fornell & Larcker, 1981). Variance inflation factors for all constructs were below five, ruling out multicollinearity concerns (Ringle et al., 2015). By validating that the interaction term between the predictors and their log transformation was significant, we ensured that our outcome variable had a linear relationship with our continuous predictors (Hosmer et al., 2013). Further, we ensured that the moderator variables of our model were not significantly correlated with the dependent variable (Hosmer et al., 2013). Since the scales demonstrated sufficient internal consistency, we used the averages of all latent variables to form composite scores for subsequent statistical analysis. We tested whether our manipulations were noticeable and successful by including a manipulation check in our experiment: Right after the first task, participants were asked, “Based on what you’ve read, what percentage of user input does [name of the bank] chatbot understand on average?” Participants then had to choose an answer from a multiple-choice list comprising the treatments: No information was given on this/Don’t know, 60%, 95%, a lot, almost all. Participants who failed to answer the manipulation check correctly were immediately excluded from the experiment.

## Results

### Data collection

We used an expert panel (comprising IS researchers) and performed a pilot test to validate the messages and instruments prior to the final data collection. We made minor changes to the experiment instructions and to some of the wording. We conducted the final web-based experiment in September 2020 using Qualtrics. Like a growing number of IS papers, we recruited participants from the crowdsourcing platform Prolific Academic (e.g., Adjerid et al., 2018; Betzing et al., 2020; Teubner et al., 2019). Prolific is, in most respects, similar to Amazon’s Mechanical Turk platform, except that participants on that platform only participate in academic research. Prolific has been found to produce high-quality data in terms of participants’ attention, reliability and reproducibility (Peer et al., 2017). We incentivized participants by offering them a maximum payoff of 1.56 GBP, fulfilling the requirements of a consequential incentive-compatible experiment. However, since we were not up front with the participants about the actual number of task-solving rounds (six rounds were announced in the study introduction, whereas we only conducted three rounds of task-solving), we gave all of them the maximum payoff after they had been debriefed. All participants were based in the UK. The sample for the study was drawn from the target population of the general adult public and specifically constructed to be representative of UK population in terms of age, ethnicity and gender (see Table 3); sample demographics closely resemble that of the UK population (Office for National Statistics, 2016).

**Table 3:** Sample Demographics

Demographics		Frequency	Sample (%)	U.K. population (%)
**Gender**	Male Female	277 2815	49.6% 0.4%	49.0% 51.0%
**Age**	18 to 27 28 to 37 38 to 47 48 to 57 > 57	186 137 72 76 58	33.3% 24.6% 12.9% 13.6% 15.6%	17.7% 17.7% 18.7% 16.7% 29.3%
**Ethnicity**	White Mixed Asian Black Other	463 16 43 28 8	83.0% 2.9 % 7.7% 5.0% 1.4 %	76.0% 4.3% 9.7% 6.7% 3.3%

The 296 participants who failed the two manipulation checks or did not complete the survey were automatically excluded from the study. In addition, we excluded six responses that indicated technical problems with the CA or that the study’s dissimulation had been detected, by screening the feedback form at the end of the survey. We also excluded participants who failed an attention check, asking the participants for their potential incremental payoff (which was set as 0.26 pence/task). We collected 564 responses in total. However, the final number after cleaning was 558.

### Hypotheses testing

To test the main effect and moderation effect hypotheses, we conducted a logistic regression (logit) analysis on the dependent variable *actual discontinuance* (see Table 4). Concerning the hypothesized main effects, the results demonstrate that perceived performance negatively impacted the dependent variable (β = -0.549, p = 0.004), thus supporting hypothesis 1. Users that perceived the CA’s performance as good after a CA response failure were less likely to discontinue its usage (Odds Ratio = 0.578). Second, hypothesis 2 was supported through a significantly negative impact of trusting beliefs on actual discontinuance (β = -0.611, p = 0.004), indicating that users who believed the CA to be trustworthy after the response failure were less likely to discontinue its usage (Odds Ratio = 0.543).

**Table 4:** Results of logistic regression on dependent variable actual discontinuance in relation to control group (no-treatment)

			95% C.I. for Odds Ratio	
	β	Odds Ratio	Lower	Upper
Constant	1.332	3.790		
Perceived Performance	-0.549**	0.578	0.396	0.843
Trusting Beliefs	-0.611**	0.543	0.358	0.824
Manipulations				
Quantitative/Low Performance Level	0.217	1.242	0.924	1.670
Qualitative/Low Performance Level	0.049	1.050	0.817	1.351
Quantitative/High Performance Level	-0.020	0.981	0.763	1.260
Qualitative/High Performance Level	0.005	1.005	0.775	1.304
Trusting Beliefs X Quantitative/Low Performance Level	0.159	1.173	0.738	1.864
Trusting Beliefs X Qualitative/Low Performance Level	-0.617*	0.539	0.292	0.996
Trusting Beliefs X Quantitative/High Performance Level	-0.028	0.972	0.651	1.452
Trusting Beliefs X Qualitative/High Performance Level	-0.264	0.768	0.502	1.176
Perceived Performance X Quantitative/Low Performance Level	-0.519*	0.595	0.372	0.953
Perceived Performance X Qualitative/Low Performance Level	0.386	1.470	0.890	2.428
Perceived Performance X Quantitative/High Performance Level	-0.028	0.972	0.641	1.475
Perceived Performance X Qualitative/High Performance Level	-0.058	0.944	0.639	1.395
Controls				
User’s Expertise	-0.181	0.834	0.646	1.076
Degree of Realism	-0.037	0.963	0.821	1.130
Personal Innovativeness	0.173	1.189	0.953	1.483
Age	-0.007	0.993	0.934	1.057
CA Usage	-0.095	0.910	0.760	1.089

Note: n = 558, R² = 0.236 (Cox & Snell), 0.325 (Nagelkerke), Model χ2 (1) = 150,000, *p < .05; **p <.01

We suggested in hypothesis 3a that, in contrast to high performance information, the presentation of low performance information would moderate the effect of the perceived performance on actual discontinuance to a greater extent. This is supported by our results: A high performance level didn’t result in any significant interaction effects, whereas the interaction between quantitative/low performance information and perceived performance on actual discontinuance was significant (β = -0.519, p = 0.031). Similarly, hypothesis 3b is supported by the significant interaction effect between qualitative/low performance level and trusting beliefs on actual discontinuance (β = -0.617, p = 0.049), while other interaction results were insignificant.

Hypothesis 4a is supported by the results of the logistic regression, as the interaction effect between perceived performance and the quantitative performance information on actual discontinuance was significant (β = -0.519, p = 0.031), while other manipulated interactions with perceived performance didn’t show significant results. Hypothesis 4b is supported as the results show a significantly negative interaction effect between trusting beliefs and qualitative performance information on actual discontinuance (β = -0.617, p = 0.049). The support of hypotheses 4a and 4b shows that the manipulation of the linguistic form of the performance information in an inoculation message influences the route of processing (peripheral vs. central) as posited by the ELM.

All in all, in interaction with the quantitative/qualitative linguistic form, only a low performance level served as a significant moderator of the effects of the dependent variables on actual discontinuance. The linguistic form determined how the treatment was processed by the user, indicating significant results only for the central/peripheral route of information processing.

## Discussion

This study examines the use of inoculation messages as a measure to prevent users’ discontinuance of a CA following response failure. First, we were able to confirm the basic assumption of our study that a CA response failure has a negative impact on user perception in a service context, which is consistent with previous research highlighting the need for response failure-free CAs (Diederich et al., 2021; Følstad & Taylor, 2020; Gnewuch et al., 2017). Further, our results demonstrate that inoculation messages containing low performance information (vs. high performance information) about the CA alleviate the effects of perceived performance and trusting beliefs on actual discontinuance. Hence, in the case of a CA response failure, users who were sent an inoculation message at the beginning of their CA interaction were less likely to discontinue using the CA. When an inoculation message containing low performance level information was sent, the strongly negative relationship between perceived performances, trusting beliefs, and actual discontinuance was mitigated. Therefore, our results provide a response to recent calls to investigate measures to mitigate CA response errors (Diederich et al., 2021).

In addition, we show that the basic principle of inoculation messages as described in other research domains such as political and corporate communication, namely preparing individuals for impending negative events through inoculation messages, is also applicable in the domain of individual IS adoption decisions and can thus be used for specific usage scenarios (such as a CA response failures) (Compton & Ivanov, 2013; Wan & Pfau, 2004). Depending on the linguistic form of the performance information (quantitative vs. qualitative), users processed the “inoculation effect” through different cognitive routes: A quantitative form moderated the effect of perceived performance, whereas a qualitative form moderated the effect of trusting beliefs. With our results, we add further evidence for the previous findings that quantitative linguistic forms are processed more intentionally and analytically than qualitative ones, taking up more mental resources and corresponding to the central route of information processing of the ELM (Childers & Viswanathan, 2000; Liu et al., 2021). Moreover, we could also confirm previous findings on the peripheral route of the ELM: a qualitative linguistic form is processed more intuitively and activates fewer mental resources (Ayal et al., 2015; Liu et al., 2020; Tzelgov et al., 1992). Further, our results demonstrate that, in contrast to prior research that focused on ex-post strategies to CA response failures (Ashktorab et al., 2019; Følstad & Taylor, 2020; Lee et al., 2010; Medhi Thies et al. 2017), measures taken before the response failure can help alleviate negative effects on user behavior. More importantly, the alleviation effect of inoculation messages is not limited to users continuing to use the CA, as it has an effect before the user decides to discontinue. Hence, the risk of losing contact with the user after a CA response failure can be ruled out. Overall, these results have several theoretical and practical implications, which we discuss below.

### Theoretical implications

Our work takes up the call of Diederich et al. (2020) to further user-centric investigation of CA response failures. To this end, our study empirically investigates the impact of CA response failures on users’ perceptions and behavior, while proposing and evaluating an alleviation mechanism (i.e., inoculation messages). Our study represents a first step in addressing two major limitations of user-centric CA literature. Firstly, the focus of prior IS research on response failure-free CA interactions is not congruent with contemporary performances of CAs, thus limiting the transferability of results. Further, neglecting response failures in CA research could slow down the dissemination of CA technology because of the unrealistic expectations arising from this research as well as a lack of relevant research-based design recommendations. Taking CA response failures into account, our study offers a cost-efficient measure to alleviate the negative effect of a response failure on users’ continuing usage.

Secondly, those studies that address CA response failures focus on ex-post alleviation mechanisms, thus not providing opportunities to preemptively address users so that they will continuing using the CA after a response failure (Ashktorab et al., 2019; Følstad & Taylor, 2020; Lee et al., 2010; Medhi Thies et al. 2017). Implementing measures prior to actual CA response failures is important because users often discontinue their usage right *after* a CA response failure, which would make it impossible to present the (ex-post) measure to the user. Our study shows that inoculation messages, presented ex ante, lessen users’ discontinuance.

Second, we contribute to CA research by presenting a measure that has the potential to make contemporary chatbots more successful (Janssen et al., 2020). Our work shows that inoculation theory, and inoculation messages in particular, is effective for fostering user acceptance after a CA response failure. Drawing on widely neglected inoculation theory, we designed system messages that significantly alleviate the discontinuance effect of a CA response failure. To ensure the effectiveness of inoculation messages, we propose a “contextually specific” design based on a two-sided inoculation message, featuring CA performance information (Wright et al., 2014, p. 398). Most CA literature focuses on the important question of how to improve the utilitarian value of CA components (e.g., anthropomorphism) in relation to user perception and acceptance (Diederich et al., 2020; e.g., Zhang et al., 2012). However, research focusing on short-term oriented and immediate improvements is necessary to ensure future relevance of CA technology by providing practitioners with go-to measure to improve their CA. Hence our study offers a complementary perspective on CA research, ensuring long-term relevance while focusing on short-term success.

In addition to the literature on CAs, our work contributes to the ELM literature by examining the role of inoculation messages on individual’s information processing in the context of CA response failures. To foster specific usage behavior, prior to an initial usage, previous ELM literature highlighted the importance of methods like education and guidance (Ölander & Thøgersen, 2014; Shleifer, 2012). However, these methods are limited since they convey persuasion through the central processing of presented information. This requires that the user is capable and motivated so that central processing can be activated. Due to these limitations, prior literature called for research on additional tactics, including evidence on which cognitions they entail, and how they could be selectively evoked to foster usage (Ferratt et al., 2018). We showed that the differentiated usage of performance information within inoculation messages evokes a central, peripheral respectively, route of processing of the inoculation message, indicated by a moderator effect on the respective route. Hence, we contribute to ELM literature by providing a novel tactic to foster usage, while highlighting that quantitative/qualitative performance in inoculation messages will be processed on the central/peripheral route.

### Practical implications

Our paper has important implications for CA providers, such as platform providers or firms with direct online customer interactions given the importance of CAs in customer service. First, we recommend the use of inoculation messages if there is a risk of CA response failure. By showing that we can alleviate the negative effects of response failures on users’ continuing usage of the CA, we provide evidence that alerting users to the possibility of CA response failures in combination with refutational pretreatments will help users stay with the CA right after a CA response failure rather than discontinuing usage. While the refutational pretreatment should address the CAs’ performance, performance information should be regarded carefully because, for a high performance level condition, the positive effect of an inoculation message is offset: In the moment of a response failure, high performance level information can serve as an indicator for users to discount the CA’s credibility, as they might perceive this information to be incorrect. Thus, providers who attribute a high performance level to their CAs might not see an alleviative effect of inoculation messages. Providers of low performance level CAs, on the other hand, should build on our results and implement inoculation messages prior to CA usage to lessen the rate of users discontinuing their usage after a CA response failure.

Further, we found that qualitative performance information (e.g., “almost all”) are processed differently by users, compared to quantitative performance information (e.g., “98%”). While a quantitative linguistic form affects the perceived performance/discontinuance relationship, a qualitative form affects the trusting beliefs/discontinuance relationship. We recommend providers to choose between a qualitative linguistic form and a quantitative one, depending on the context of the CA and the product/service they are offering. The qualitative form is best used. In fast-paced consumption contexts, such as convenience products (e.g., food), since these users are cognitively less involved in the buying decision (Franke et al., 2004). The qualitative form is processed intuitively and therefore does not require any additional working memory on the part of the user. It is best used in contexts where consumption is less frequent and where users are heavily invested in the purchasing process, as in the case of shopping products (e.g., cars).

Although inoculation messages alleviate the negative effects of CA response failures, thus fostering adoption, providers’ ethical evaluation is critical here. The usage of inoculation messages needs to be assessed regarding the ethical principles (i) public interest, (ii) honesty and accuracy, and (iii) quality of the artifact, as stated by Myers and Venable (2014). (i) This means that before implementing inoculation messages, CA providers need to critically consider which benefits or harms may result for all stakeholders. Instead of the primary goal being to persuade customers to use a service and, if necessary, even to conceal or gloss over any undesirable consequences, inoculations messages should primarily serve to inform customers about the expected functionality and possible consequences so that they can form an objective opinion. (ii) In addition, CA providers should be honest and accurate in their assessment of CA performance when providing inoculation messages. This is critical to inoculation messages, because an important part of the messaging (i.e., refutational pretreatment) is built on the disclosure of the CA's actual performance. If an accurate estimate of the CA's performance is not possible, providers should estimate the performance to the best of their knowledge and belief. (iii) In addition, before using inoculation messages, providers should ensure that they have reached the limit of their capacity in terms of increasing the security and functionality of the CA. Thus, it should be ensured that the CA has undergone a structured product development process and no avoidable defects remain before its integration in actual customer processes.

### Limitations and suggestions for future research

Based on the paper’s limitations, this paper offers several suggestions for future research focusing on the design of CAs, which may help alleviate the negative impact of response failures on users’ actual continuance.

First, our study employed a simplified example of a text-based CA and response failures. Hence, future research should aim to validate our findings in a real-life setting, such as a field study. Future studies could compare the impact of inoculation messages on actual discontinuance across several CA user interfaces that differ in levels of anthropomorphism (e.g., voice interface, avatars). In a field study, particular attention should be paid to the role of typical distractions and breaks in CA conversations (e.g., notifications, external influences), which we deliberately minimized by providing a controlled, basic web interface for the conversation. Second, due to our methodological design, we revealed only the effects based on operationalized manipulations, but investigating other forms of CA response failures and inoculation messages would be interesting. Researchers could compare timing effects of a response failure, varying the moment of a response failure (e.g., early vs. late), preferably in longitudinal studies where long-term effects of CA response failures on usage behavior could be investigated. Third, our sample consisted of only UK participants. Thus, future research would benefit from investigating the effects of inoculation messages in different cultural contexts. Fourth, our manipulations focused on text-based inoculation messages, but researchers could investigate potential interactions with other technology-mediated forms of designing information (e.g., visualizations, animation).

We also provide suggestions for future research that go beyond the paper’s limitations: Firstly, we focused on customer service, which is considered one of the most important application areas of CAs. While customer service has previously been used in CA literature to derive generalizable results (e.g., Adam et al., 2021; Diederich et al., 2019c), user motivations, goals and behaviors may differ across contexts. In particular, CA users in mandatory usage contexts (e.g., human resources departments), might perceive CA response failures and inoculation messages differently. Secondly, future research should investigate the role of individual peculiarities of the CA provider. Jiménez-Barreto et al. (2021) found that the perception of a hotel brand (sincere vs. exciting) influenced the costumers’ processing of COVID-19 cleaning messages, indicating that contextual factors can influence the processing of messages. Regarding inoculation messages, future research should investigate whether perceptions of CA providers influence the perception of inoculation messages and their effectiveness. Finally, since CAs are a relatively new implementation, researchers should investigate changes in user behavior and examine how these changes relate to system-specific variables and context-dependent factors. CAs have only recently attracted a lot of interest from businesses, and it is expected that many more CAs will be implemented in the near future. This means that users can get used to the system’s peculiarities and react differently over time once they are familiar with the system’s peculiarities.

## Conclusion

CAs have seen substantial improvements in recent years. However, they are still often unable to provide meaningful responses to users’ requests. Such CA response failures are problematic for their success and diffusion because they can cause a loss of trust in the performance of the system among users, with the possible result of usage discontinuance. In this study, we conducted a randomized online experiment to show that inoculation messages, presented to users at the beginning of a CA interaction, can alleviate the negative effects of a response failure. Particularly, this alleviative effect was present if the CA was described as low performance in the inoculation message. For CAs that were described as high performance, no alleviative effect could be shown. Further, our results indicate that the linguistic form of the performance information influences the type of cognitive processing users employ. Overall, our findings contribute to CA literature by (1) highlighting hitherto neglected user perspectives on CA response failures and (2) proposing a way to foster the long-term success of CAs. Practitioners can use our results as a guide in implementing cost-effective measures (i.e., inoculation messages) to reduce users’ discontinuance in the event of a CA response failure. We hope that our study will be the impetus for future research on alleviating the negative effects of CA response failures in and beyond electronic markets and customer self-service contexts.

## Figures and Tables

**Appendix 1:**
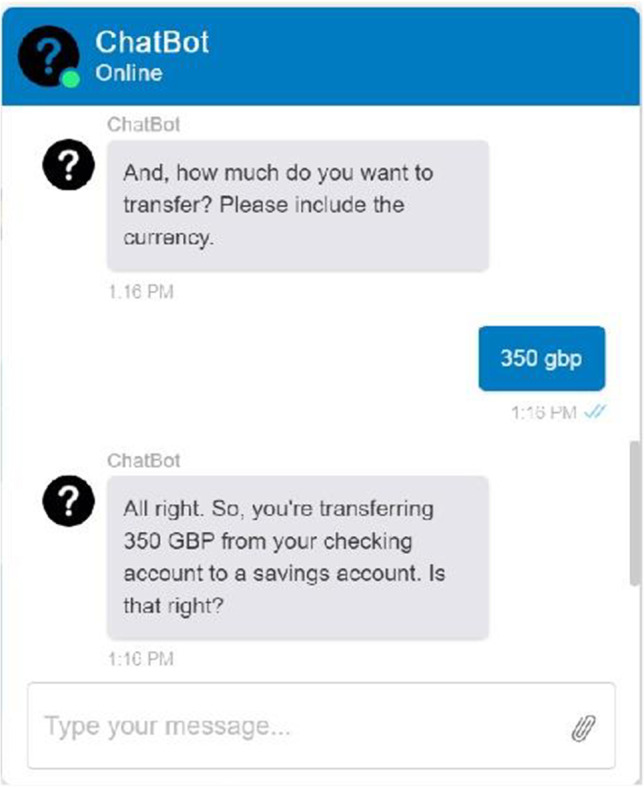
Screenshot of the chatbot interaction interface

**Appendix 2:**
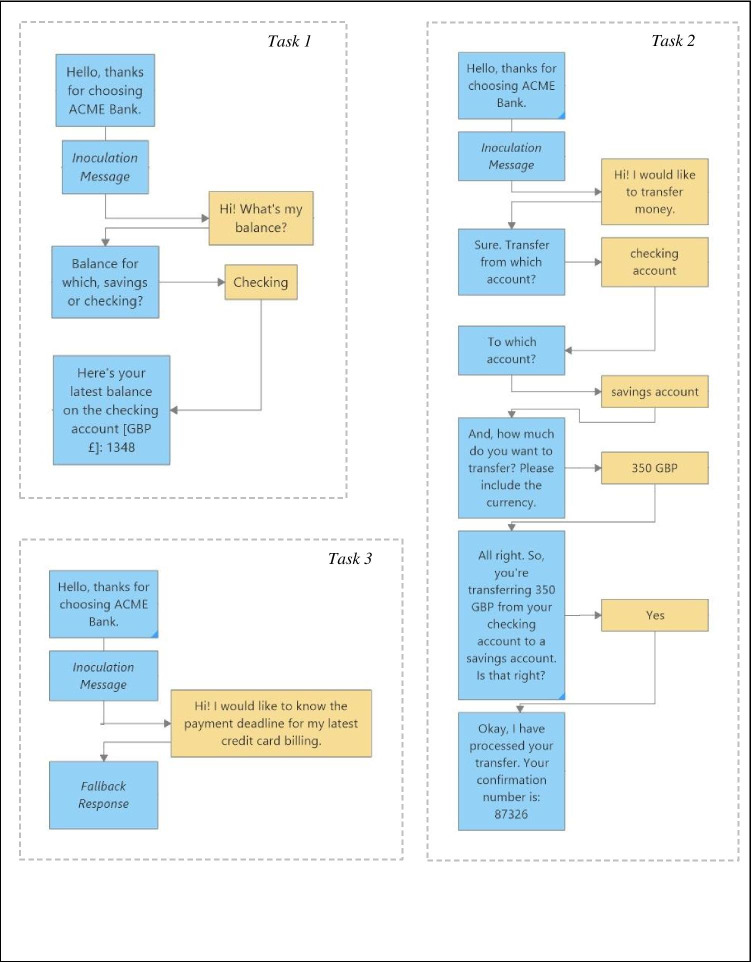
Dialogue graphs of conversations for task-solving

## Footnotes

1 Conversational computing platforms (e.g., Google Dialogflow, IBM Watson Assistant) enable sophisticated chatbot solutions that allow for effortless implementation of anthropomorphic features, even for novice users or non-developers (Diederich et al., 2019b).

2 Standard, contemporary platforms for CA design (e.g., Google Dialogflow) do not support advanced visual design features such as emojis, colors or pictures.
